# A Review of Artificial Intelligence Models for Detecting Breast Arterial Calcification on Mammograms and Their Clinical Implications

**DOI:** 10.7759/cureus.86894

**Published:** 2025-06-27

**Authors:** Suchit Chidurala, Parsa Charkhchi, Raajkiran Komirisetty, Keola Ching, Kyra Rozanitis, Tony Jha, Varshaa Koneru, Kal L Clark

**Affiliations:** 1 Long School of Medicine, University of Texas Health Science Center at San Antonio, San Antonio, USA; 2 Radiology, University of Texas Health Science Center at San Antonio, San Antonio, USA

**Keywords:** artificial intelligence in radiology, breast arterial calcification, cardiovascular disease risk, machine learning (ml), preventative mammogram

## Abstract

Breast arterial calcification (BAC), traditionally regarded as an incidental mammogram finding, is now recognized as a clinically significant marker associated with cardiovascular disease (CVD), particularly in women. Despite this association, BAC remains underreported in clinical practice due to the lack of standardized screening protocols and the manual burden of identification. With CVD being the leading cause of mortality among women worldwide and traditional risk calculators often underestimating female cardiovascular risk, the potential of BAC as a surrogate biomarker is increasingly being explored. Mammography, already a widely used screening tool for breast cancer, offers an opportunity to identify BAC and thereby enable dual-purpose screening for both breast and cardiovascular health.

Recent advancements in artificial intelligence (AI) and machine learning (ML), particularly in deep learning models such as convolutional neural networks (CNNs), have shown tremendous promise in detecting and quantifying BAC with high accuracy. Models such as DU-Net, difference-of-Gaussian generative adversarial network (DoG-GAN), and Simple Context U-Net (SCU-Net) utilize U-Net architectures optimized for segmentation and demonstrate performance metrics that rival or surpass human experts. Other approaches, including hybrid models, transfer learning, and ensemble methods, have also achieved strong diagnostic metrics, improving the reliability and scalability of BAC detection. This review consolidates findings from recent studies and technical innovations, evaluating various ML algorithms and their applications in automating BAC identification. In doing so, it highlights the potential of AI to address the long-standing challenge of underreporting and inconsistent quantification of BAC.

The clinical implications of AI-enhanced BAC detection are significant. Accurate, automated identification of BAC can improve cardiovascular risk stratification, especially in women whose disease may otherwise go unnoticed by traditional tools. Moreover, at a population level, integrating BAC detection into routine mammogram workflows could yield substantial public health benefits, enabling earlier interventions and reducing overall healthcare costs. By consolidating current models and emphasizing the need for standardized reporting, this review aims to support the integration of AI-based BAC detection into routine clinical practice, thereby enhancing both diagnostic accuracy and preventive care for cardiovascular disease.

## Introduction and background

Mammography: A window into vascular health

Mammograms are chest X-rays typically used for breast cancer screening. This non-invasive procedure involves compressing the breast between two plates, followed by an X-ray [1]. Studies have shown that mammograms reduce breast cancer mortality by 13%-17% in women aged 50-69, and the US Preventive Services Task Force recommends biennial screening for women aged 40-74 years [2,3]. Mammograms can reveal findings such as masses, architectural distortion, and asymmetries. Calcifications appearing as white spots or flecks are calcium deposits categorized as macrocalcifications or microcalcifications. Macrocalcifications, appearing as large white dots, are generally benign, whereas microcalcifications, appearing as fine white specks, can indicate pathological processes such as inflammation or neoplastic lesions based on their size, shape, and distribution [4].

Breast Arterial Calcification Assessment Criteria

Breast arterial calcification (BAC) is another type of calcification detectable via mammograms. Studies have linked BAC with chronic diseases such as coronary artery disease (CAD) and diabetes, particularly in older patients [5]. BAC appears as a radio-opaque, tram-track configuration paralleling the vessel wall [6]. Breast Imaging Reporting and Database System (BI-RADS) guidelines leave BAC reporting to the interpreter’s discretion, resulting in underreporting [7]. Furthermore, while it is biologically plausible that the extent of BAC correlates with the magnitude of cardiovascular risk, current research is limited by the lack of standardized quantitative reporting methods. Therefore, standardizing BAC reporting could improve cardiovascular disease (CVD) risk assessments, given BAC’s association with CVD risk factors. In fact, Suh and Yun proposed a scoring method for assessing BAC severity using three variables (number, length, and density of calcifications) that enables categorization into three severity groups and could standardize BAC reporting [8]. A summary of this assessment is given in Table 1. Better standardizing the reporting of BAC using the described system has clinical significance due to the association between BAC and cardiovascular (CVD) risk.

**Table 1:** Summary of Proposed BAC Assessment Criteria BAC: breast arterial calcification Source: [8]

Assessment criteria	Description
Number of calcifications	0-6 vessels (anything over 6 is counted as 6)
Length of calcification	0-3 based on the longest calcification
Density of calcification	0-3 based on the densest segment of calcification
Overall sum	0 (none)
	1-6 (mild)
	7-12 (severe)

Breast arterial calcification: Its connection to cardiovascular disease

Clinical Significance

BAC is easily detected on mammograms and has a prevalence ranging from 10% to 12% in healthier populations, increasing to 60%-70% in women over 70 years [8]. Literature reviews report a positive correlation between BAC and increased CVD risk, with BAC presence associated with a 1.59-fold higher risk of CAD [8-10]. Most studies to date evaluate BAC as a binary variable (present or absent) rather than quantifying its extent. This has prevented a detailed analysis of how varying degrees of BAC correlate with the severity of cardiovascular disease. Nevertheless, the presence of BAC is still a strong indicator of a higher risk of CVD, with many studies linking BAC to a higher risk of CVD events and an overall higher risk compared to the general population. Because of this association, identifying incidental BACs can be a potentially efficient means to better calculate cardiovascular risk in women.

CVD, including CAD, is the leading cause of mortality and disability-adjusted life years (DALYs) worldwide, accounting for nearly seven million deaths and 129 million DALYs annually [11]. CAD is due to atherosclerotic plaque buildup in coronary arteries, decreasing the blood flow to the heart. These plaques are extremely prevalent, and calcification of these plaques is present in 50% of individuals aged 40-49 and 80% of individuals aged 60-69 [12,13]. In the USA specifically, CVD is the leading cause of death, costing $252.2 billion from 2019 to 2020 [14]. Because of the significant burden CVD presents, it is essential to estimate CVD risk to direct preventative measures.

Despite estimating CVD risk, cardiovascular mortality has recently increased by 1% annually in women aged less than 55 years, and about 20% of ischemic heart disease events in women occur in the absence of the traditional CVD risk factors [15]. Furthermore, when coronary artery calcifications are detectable, women also have a 1.3 times higher hazard for cardiovascular death compared to men [16,17]. This sex difference in CVD mortality may be due to the underestimation of CAD risk in women and misclassification of women with subclinical disease as low risk through traditional CVD risk assessment tools such as the Framingham Risk Score [18]. Modifying current CVD risk assessment tools can better estimate risk in women, as the Multi-Ethnic Study of Atherosclerosis reported that the addition of coronary artery calcification scoring improved risk stratification in women compared with traditional tools [19]. Additionally, Margolies et al. found that BAC is highly sensitive to the presence of coronary artery calcification, and severe BAC was predictive of coronary artery calcification, with a predictive value that was equivalent to, and even additive in, identifying high-risk women compared to traditional CVD risk assessment tools [20]. Therefore, with millions of mammograms conducted every year, identifying BAC on these scans could serve as an efficient preventative health method to help mitigate the sex difference in CVD mortality by improving CVD risk calculation in women, as women with BAC have an increased risk for CVD events, even after adjusting for age and traditional risk factors [21]. Lastly, by identifying BACs on mammograms, further scans for calcification and more invasive methods to calculate CVD risk can be avoided, saving patients from radiation exposure, costs, and time.

Pathophysiology

Vascular calcifications are critical markers and contributors to CVD pathogenesis, arising from metabolic, genetic, and environmental factors. Calcification results from active processes involving osteogenic differentiation of vascular smooth muscle cells (VSMCs), inflammatory pathways, oxidative stress, and lipid accumulation. The pathophysiological interplay involves complex mechanisms, including inflammatory processes, oxidative stress, and dysregulation of mineral metabolism, which collectively foster the deposition of calcium phosphate in the vascular matrix [9,22]. Manifesting as hardened deposits in the arterial walls that compromise vascular elasticity and function, vascular calcification predisposes individuals to atherosclerosis and CAD, significantly impacting the CVD risk profile.

Historically, vascular calcification was considered a passive process, resulting from calcium and phosphate ions exceeding solubility in tissue fluid, thereby inducing the precipitation and deposition of hydroxyapatite crystals [23]. However, increasingly strong evidence has supported that this process has a much more complex and active pathogenesis through different pathways depending on the location of calcification: medial or intimal [24,25]. Mönckeberg medial calcification primarily affects the tunica media of arteries in individuals with long-standing diabetes, chronic kidney disease, and hypercalcemia. This is the type of calcification detected by mammograms. Altered intracellular signaling pathways and calcium-sensing receptors cause VSMCs in the tunica media to differentiate into osteoblast-like cells, causing Mönckeberg sclerosis [24,25]. It is independent of atherosclerosis as it does not involve lipid deposition or inflammatory processes. While Mönckeberg sclerosis typically occurs in peripheral arteries, it can still occur in any artery and lead to CVD [26]. In fact, BAC is a type of Mönckeberg medial calcification that occurs in the mammary arteries.

In contrast, intimal atherosclerotic calcification (IAC) occurs in the innermost layer of blood vessels: the tunica intima [27]. IAC always begins with atherosclerosis. Once a plaque has formed, de novo calcification in IAC is due to two main mechanisms, with the first being the loss of mineralization inhibitors such as osteopontin, fetuin, and γ-carboxyglutamic acid Gla protein [28]. The loss of mineralization inhibitors is due to the dysregulated balance between promotion and inhibition of calcification in chronic kidney disease, diabetes mellitus, atherosclerosis, and as a consequence of aging [29]. In addition, IAC also involves VSMC differentiation into osterochondroblast-like cells, which is triggered by inflammation present [30]. Lipid accumulation causes this inflammation within the intima, particularly oxidized low-density lipoprotein (LDL). Inflammatory cells release matrix vesicles and apoptotic bodies during cell death, which serve as nucleation sites for hydroxyapatite, creating pro-osteogenic conditions for VSMC differentiation regulated by bone morphogenetic protein (BMP) [31]. BMPs regulate osteoinduction in calcifying vascular cells and activate osteoblastic genes such as Msx-two and Runx/Cbfa1, which are essential for osteoblast differentiation [31,32]. This process is further exacerbated by oxidative stress and inflammation, where cytokines such as interleukin-6 and tumor necrosis factor-alpha play crucial roles [33]. Coronary artery calcification found in CAD is almost always due to this type of calcification. The prognostic value of coronary vascular calcifications cannot be overstated, as it is a strong predictor of CVD risk since calcified plaques can contribute to the instability of atherosclerotic lesions, increasing the risk of acute cardiovascular events [34]. With the high sensitivity of coronary artery calcification in women with BAC, this finding can be used as a preventative measure to better estimate CVD risk in women and reduce the sex difference in CVD mortality.

In summary, the detection of BAC on mammograms has become increasingly important due to its role in early screening and risk stratification for CVD. Despite advances in technology, the manual identification of these calcifications remains a time-intensive and subjective process that is often underreported due to a lack of standardized guidelines. This review examines the role of machine learning and artificial intelligence (AI) in automating and streamlining the detection of BAC, focusing on existing models, their methodologies, and their clinical implications in enhancing diagnostic capabilities. By synthesizing findings from recent studies, this work aims to identify gaps in current approaches and highlight opportunities for future innovation.

## Review

Traditionally, radiologists would manually examine scans for abnormalities, such as masses and calcifications, and classify their findings using the BI-RADS. Occasionally, multiple radiologists may review a scan to ensure accuracy. If a BAC was found, it may be noted on the report. However, since there are currently no guidelines regarding the reporting of BAC, it is often underreported on radiology reports. The development of computer-aided detection systems has enhanced this process by algorithmically highlighting important areas in the images to guide radiologists [35].

Recent advancements involve deep learning algorithms, which use machine learning (ML) to process images similarly to the human brain. These algorithms require large labeled datasets of mammogram images for training to classify abnormalities, including masses, calcifications, and architectural distortions. Convolutional neural networks (CNNs) are particularly suited for image analysis. They scan two-dimensional (2D) images for features using a convolutional filter, combining them through pooling layers for object detection or classification. This is done by fine-tuning previously trained models, known as transfer learning, which reduces the time and resources needed for training. Ensemble methods, which aggregate outputs from different convolutional networks, can improve the overall accuracy and reliability of the models [36].

ML models are increasingly recognized for their potential to enhance medical imaging practices, particularly in the field of mammography, where they have shown potential to detect early signs of vascular calcification. Specifically, convolutional neural networks (CNNs) effectively interpret medical images by learning deep features without preprocessing steps such as segmentation or feature extraction. However, their success depends on large annotated training sets, which are often unavailable.

U-Net is a convolutional neural network architecture designed for biomedical image segmentation. It features a U-shaped structure with an encoder-decoder path and skip connections that allow for precise localization by combining contextual and spatial information. U-Net excels in segmenting medical images, even with relatively small annotated datasets, due to its efficient architecture and high accuracy [37].

U-Net-based CNNs

DU-Net is a variation of the U-Net model enhanced with dense connectivity to improve its capability in detecting BACs in mammograms. This model has been shown to surpass the diagnostic accuracy of both human experts and other ML models [38]. Alamir et al. introduced a method utilizing a difference-of-Gaussian generative adversarial network (DoG-GAN) for the segmentation of BACs in mammograms [39]. This method creatively combines a multi-scale Difference of Gaussian pyramid with a U-net generator within the GAN framework. This approach is specifically designed to enhance detail recognition across various scales and effectively capture edge information, leading to improved segmentation of BACs. The performance of the DoG-GAN model has been rigorously tested on synthetic 2D images from digital breast tomosynthesis examinations and has demonstrated superior results compared to existing methods [39].

**Figure 1:**
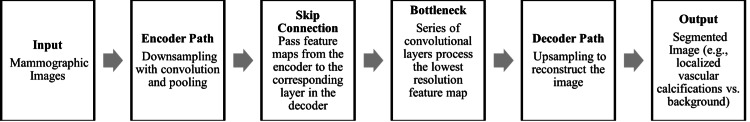
U-Net-Based CNN Flowchart A summary of the U-Net-based CNN algorithm. CNN: convolutional neural network This figure was created by the authors.

In another study, Guo et al. introduced the Simple Context U-Net (SCU-Net), a new, lightweight vessel segmentation approach suitable for handling the large image sizes typical of mammograms [40]. This model employs a patch-based training strategy, allowing for the stitching together of patch-wise results to achieve a comprehensive whole-image analysis. To further quantify the calcifications detected, Guo et al. tested five quantitative metrics that proved highly effective [40]. These metrics showed a correlation of over 95% with standard measures on breast CT scans, including calcified voxels and calcium mass. The results from this study suggest that SCU-Net could serve as a valuable clinical tool for monitoring the progression of BAC, providing robust segmentation performance and reliable quantification of calcifications [40]. Figure 1 summarizes the underlying algorithm used by U-Net-based CNNs.

Non-DU-Net-based CNNs

Wang et al. developed a CNN to quantify BACs by classifying individual pixels as either BAC regions or not [41]. Using 840 full-field digital mammograms, they achieved a free-response receiver operating characteristic (ROC) curve comparable to expert radiologists, underscoring the efficacy of such models [41].

Expanding the application of deep learning in mammography, Mobini et al. utilized a deep convolutional neural network to automatically detect and quantify BAC from mammograms [42]. This retrospective study involved multiple readers labeling four-view mammograms as either BAC positive or negative. Using a pre-trained Visual Geometry Group (VGG) 16 model, the researchers refined the system to discriminate effectively between BAC-positive and BAC-negative mammograms, based on several key performance metrics, including accuracy, F1 score, and area under the receiver operating characteristic curve (AUC-ROC). The CNN showed impressive results across training, validation, and test sets, with strong correlations noted with manually measured BAC lengths [42]. This study underscores the model’s promising performance in both detecting and quantifying BAC, reinforcing the potential of deep learning applications in clinical settings.

Hybrid models

**Figure 2:**
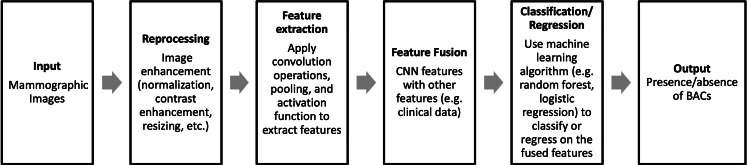
Hybrid Model Flowchart A summary of the hybrid model algorithm. BAC: breast arterial calcification, CNN: convolutional neural network This figure was created by the authors.

Songsaeng et al. employed a multi-scale architecture incorporating craniocaudal and mediolateral oblique views to classify images as either negative or positive for calcifications [43]. Their model, DLA-SE-Res2NeXt-60, which included squeeze-and-excitation (SE) and deep layer aggregation (DLA) modules with higher resolution images, achieved an 8.06% higher AUC-ROC (90.36%) compared to a traditional ResNet-50 model (82.30%) [43]. Additionally, the hybridized extreme learning machine (ELM) algorithm has also shown promising results in the detection of microcalcifications, achieving an impressive 99.04% accuracy [44]. This accuracy rate significantly outperforms that of standard classifiers such as support vector machines (SVM) and Naïve Bayes algorithms [44]. The algorithm used by hybrid models is summarized in Figure 2.

Transfer models

**Figure 3:**
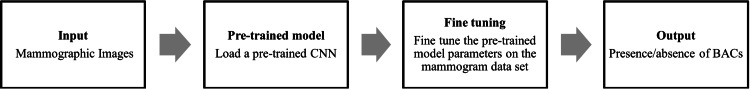
Transfer Model Flowchart A summary of the transfer model algorithm. BAC: breast arterial calcification, CNN: convolutional neural network This figure was created by the authors.

Khan and Masala employed a transfer learning approach to effectively classify BACs into four severity grades, achieving an impressive 94% accuracy with pre-trained deep learning models such as VGG-19, ResNet50, DenseNet121, InceptionV3, and MobileNet [45]. Each of these models brings unique architectural benefits, including efficient feature extraction and adaptability to limited computational resources, which significantly enhance their performance in clinical settings [45]. Figure 3 summarizes the algorithm used by transfer models.

Ensemble models

Sert et al. applied various preprocessing techniques, including contrast enhancement and cropping, and used a pre-trained GoogLeNet model to train various models on the Digital Database for Screening Mammography (DDSM) dataset [46]. They created ensembles of these models, achieving better accuracy (94.3% versus 93.1%), recall (94% versus 89.1%), and F1 score (94.5% versus 93.1%) compared to the best non-ensemble model [46].

**Figure 4:**
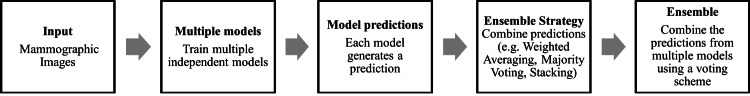
Ensemble Model Flowchart A summary of the ensemble model algorithm. This figure was created by the authors.

Similarly, Rampun et al. designed an ensemble model using various modifications of the original AlexNet and selected the three best models based on validation accuracies trained on the DDSM database [47]. The ensemble model achieved higher classification accuracy and AUC-ROC than any of the individual models (80% versus 75%-77%) and surpassed the original AlexNet [47]. The algorithm used by ensemble models is summarized in Figure 4.

Not specified models

Nerlekar et al. evaluated a deep learning algorithm named cmAngio™ by CureMetrix, which quantifies breast arterial calcification on mammograms and assesses its correlation with clinical CVD [48]. The study involved a large sample of images and included cases with confirmed CVD. The algorithm’s performance was compared against evaluations made by human readers, with any discrepancies adjudicated by a third observer. The algorithm not only achieved high accuracy but also identified BAC in cases where human readers did not, suggesting that BAC presence correlates with a higher incidence of CVD. This model is commercially available and has been cleared by the Food and Drug Administration (FDA) [48]. The model was previously trained with an 80:20 split using over 34,000 2D full-field digital mammograms and digital breast tomosynthesis mammograms obtained from multiple sites across 13 healthcare facilities in Australia, Brazil, and the USA [49].

The studies presented illustrate the transformative impact that integrating deep learning artificial intelligence models can have on clinical practice. These models not only enhance BAC reporting without increasing the clinical workload but also facilitate large-scale studies on the role of BAC as a biomarker for cardiovascular risk. This integration ultimately promotes a broader awareness of women’s cardiovascular health through improved mammographic screening, marking a significant step forward in both diagnostic capabilities and preventative care. Table 2 summarizes the different machine learning models.

**Table 2:** Summary of Machine Learning Models for BAC Detection BAC: breast arterial calcification, CNN: convolutional neural network, DoG-GAN: difference-of-Gaussian generative adversarial network, SCU-Net: Simple Context U-Net, ELM: extreme learning machine, SVM: support vector machine, FDA: Food and Drug Administration

Reference	Model name	Architecture	Key features	Dataset	Accuracy	Notes
AlGhamdi et al. (2020) [38]	DU-Net	CNN (U-Net)	Dense connectivity for enhanced accuracy	Mammograms	91.47%	Superior to human experts
Alamir et al. (2023) [39]	DoG-GAN	CNN (U-Net)	Multi-scale DoG pyramid, U-net generator	Synthetic 2D	99.69%	Enhanced detail recognition
Guo et al. (2021) [40]	SCU-Net	CNN (U-Net)	Lightweight, patch-based training	Mammograms	99.7%	Quantification metrics for BAC
Mobini et al. (2023) [42]	VGG16	CNN	Pre-trained model for BAC quantification	Mammograms	97%	Strong correlation with manual BAC length
Songsaeng et al. (2021) [43]	ResNet and ResNeXt	CNN	Multi-scale architectures, hierarchical block-wise, and layer-wise feature representation	Mammograms	84.34%	-
Melekoodappattu and Subbian (2019) [44]	Hybridized ELM	Hybrid	Outperforms SVM and Naïve Bayes	Mammograms	99.04%	Outperforms SVM and Naïve Bayes
Khan and Masala (2023) [45]	VGG-19, ResNet50, DenseNet121, InceptionV3, Mobile Net	CNN, transfer learning	Comparison of multiple pre-trained models (VGG-19, ResNet50, DenseNet121, InceptionV3, Mobile Net)	Mammograms	94%	Pre-trained models perform well in screening BAC
Sert et al. (2017) [46]	GoogLeNet	CNN, ensemble	Dilation and contrast stretching preprocessing; decision fusion by an ensemble of networks	Mammograms	94.3%	Improved preprocessing specific to BAC
Nerlekar et al. (2022) [48]	cmAngio™	Deep learning (CNN)	Commercially available, FDA-cleared	Mammograms	98%	High accuracy, detects BAC missed by humans

Accuracy, sensitivity, and specificity of various methods

The accuracy, sensitivity, and specificity of traditional methods versus ML-based methods for detecting calcifications in mammograms can vary significantly. Traditional radiologist interpretation is highly dependent on the skill and experience of the radiologist, which can lead to variability in accuracy. Furthermore, with no standardized method or requirement in reporting BAC, many radiologists do not even look for this finding on scans. ML-based methods, particularly those using deep learning and ensemble approaches, have shown potential to achieve higher and more consistent accuracy, sensitivity, and specificity in finding BAC.

For instance, Wang et al.’s CNN approach demonstrated efficacy comparable to expert radiologists, highlighting the potential of these advanced ML methods in clinical practice [41]. Furthermore, the DLA-SE-Res2NeXt-60 model by Songsaeng et al. demonstrated significant improvements in AUC-ROC compared to traditional models [43]. Similarly, the ensemble models developed by Sert et al. [46] and Rampun et al. [47] showed enhancements in accuracy, recall, and F1 scores over non-ensemble models. Overall, the integration of ML-based methods into mammogram analysis offers promising advancements in the detection of vascular calcifications, potentially leading to improved diagnostic accuracy and better patient outcomes.

Implications for population health

The potential impact of ML on population health through BAC detection is substantial. Allen et al. concluded that both BAC presence and quantity are significantly and independently associated with mortality and CVD outcomes, with BAC appearing specifically predictive of CVD risk among younger women [49]. Furthermore, Ibrahim et al. similarly found that AI detected the presence and severity of BAC and is crucial for primary CVD prevention [50]. In the USA, where approximately 75.7% of women adhere to recommended breast cancer screening guidelines, an estimated 26.8 million women over 40 are projected to undergo mammography in 2025 [51,52]. Considering a conservative 10% prevalence of BAC on mammograms, this translates to 2.68 million incidental BAC findings annually. Machine learning models, with a demonstrated specificity of 96.7% in BAC detection, could accurately identify nearly 2.6 million true positive cases each year, facilitating early intervention and risk stratification for cardiovascular disease. However, the absence of standardized quantitative reporting methods limits our ability to further stratify risk within this group. The need for a scale is clear, as it may be that some individuals within this group are at a much higher risk than others. Furthermore, the automation of identifying BAC can tremendously save costs in the healthcare system as it prevents further scans or laboratory tests needed to assess CVD risk.

Clinical relevance

BAC identified on mammograms are not only markers for potential breast pathology but also significant indicators of CVD risk in women. Recognizing BAC during routine mammography can enhance CVD risk assessment, especially in women, where traditional tools underestimate risk. This proactive approach could help mitigate the sex disparity in CVD mortality and reduce the need for invasive diagnostics. ML-powered algorithms demonstrate high accuracy, and early intervention based on ML-detected BAC could improve outcomes and yield substantial public health benefits within existing mammography frameworks. While precise life-year quantification requires further study, models show potential for extending life expectancy and improving quality of life through timely prevention. Early detection could also reduce healthcare costs by minimizing expensive treatments for advanced CAD. Validating ML models across diverse populations and integrating them with other CAD risk factors could enhance risk stratification. Future studies should create standardized tools for consistent BAC evaluation and prognostic assessment.

The potential for ML to revolutionize BAC detection and quantification aligns with the assertions by Polonsky and Greenland [53] and Daniels and Itchhaporia [54] that identifying BAC represents an untapped opportunity for simultaneous breast cancer and cardiovascular disease screening, demonstrating the urgency of developing efficient and accurate methods for BAC assessment. The current absence of standardized reporting guidelines, as they note, further amplifies the need for innovative solutions that seamlessly integrate into existing radiology workflows.

Assessing BAC could also be useful in evaluating the risk of other diseases. A meta-analysis of six cross-sectional studies revealed that the odds ratio of stroke in those with BAC versus those without BAC was 1.54 (p<0.0001) [55], indicating a significant association between BAC and stroke. Chronic kidney disease was found to be associated with increased medial BAC, indicating increased exacerbation with calcification, but not initiation [56]. Lastly, a study of 567 post-menopausal women revealed a statistically significant relationship between BAC and osteoporosis, highlighting the ability of BAC as a screening tool [57].

Current literature shows a promising slate of machine learning models for the detection of BAC to meet this need. The potential application of ML can address these critical gaps by offering a standardized, quantitative approach to BAC assessment that can easily be incorporated into mammography reports. Automating the detection and quantification of BAC alleviates the burden on radiologists and ensures that this clinically relevant incidental finding is no longer overlooked. By harnessing the power of machine learning, we can move closer to a future where the detection of BAC is not merely an incidental finding but an essential component of comprehensive patient care.

## Conclusions

BAC, once considered an incidental finding, is now increasingly recognized as a valuable marker for CVD risk in women. With mammography being a widely utilized screening tool, the incidental detection of BAC offers a unique opportunity for dual-purpose screening that benefits both breast and cardiovascular health. However, the lack of standardized reporting protocols and the variability of manual interpretation present significant barriers to its routine clinical use.

Advancements in ML and AI offer transformative solutions by enabling automated, accurate, and scalable BAC detection. A variety of models, particularly those using convolutional neural networks and ensemble techniques, have demonstrated high accuracy and potential for integration into clinical workflows. Incorporating ML-based BAC detection into routine mammography could improve cardiovascular risk stratification, especially in women who are underrepresented by traditional assessment tools. Future efforts should focus on validating these models across diverse populations and establishing standardized scoring systems to ensure consistent, reliable, and clinically actionable BAC evaluation.
